# Sexual dimorphism in up-regulation of suppressors of cytokine signaling genes in patients with bipolar disorder

**DOI:** 10.1186/s12888-019-2396-9

**Published:** 2019-12-16

**Authors:** Amir Keshavarzi, Mohammad Mahdi Eftekharian, Alireza Komaki, Mir Davood Omrani, Vahid Kholghi Oskooei, Mohammad Taheri, Soudeh Ghafouri-Fard

**Affiliations:** 1 Research Center of behavioral Disorders and Substances Abuse, Hamadan University of Medical Sciences, Hamadan, Iran; 2 Neurophysiology Research Center, Hamadan University of Medical Sciences, Hamadan, Iran; 3 Urogenital Stem Cell Research Center, Shahid Beheshti University of Medical Sciences, Tehran, Iran; 4 Department of Laboratory Sciences, School of Paramedical Sciences, Torbat Heydariyeh University of Medical Sciences, Torbat Heydariyeh, Iran; 5 Department of Medical Genetics, Shahid Beheshti University of Medical Sciences, Tehran, Iran

**Keywords:** Suppressors of cytokine signaling, Bipolar disease, Expression

## Abstract

**Background:**

Proteins encoded by *Suppressors of cytokine signaling* (*SOCS*) genes have critical roles in the regulation of immune responses. Meanwhile, several lines of evidence support the presence of immune dysfunction in bipolar disorder (BD) patients.

**Methods:**

In the present study, we assessed expression levels of *SOCS1–3* and *SOCS5* genes in peripheral blood of patients with BD and healthy subjects.

**Results:**

All *SOCS* genes were up-regulated in patients compared with healthy subjects. However, when comparing patients with sex-matched controls, the significant differences were observed only in the male subjects except for *SOCS5* which was up-regulated in both male and female patients compared with the corresponding control subjects. Significant pairwise correlations were found between expression levels of genes in both patients and controls. Based on the area under curve values, *SOCS5* had the best performance in the differentiation of disease status in study participants (AUC = 0.92). Combination of four genes increased the specificity of tests and resulted in diagnostic power of 0.93.

**Conclusion:**

Taken together, these data suggest a role for *SOCS* genes in the pathogenesis of BD especially in the male subjects. Moreover, peripheral expression levels of *SOCS* genes might be used as a subsection of a panel of diagnostic biomarkers in BD.

## Background

Bipolar disorder (BD) is a serious and insistent psychiatric disorder resulting in substantial morbidity and mortality. Among several theories for describing the principal patho-etiology of this disorder, immune dysfunction has extensively attained attention of researchers [1]. Comorbidity of BD with inflammatory diseases, dysregulation of cytokine levels in both peripheral blood and central nervous system (CNS) of patients and relapsing-remitting nature of disease are some clues that pointed out the significance of immune responses in the pathogenesis of BD [1]. Tumor necrosis factor-α (TNF-α) and soluble tumor necrosis factor receptor type 1 (sTNF-R1) are among cytokines whose levels have been consistently higher in manic patients compared with both healthy individuals and euthymic patients [2].

Suppressor of cytokine signaling (SOCS) proteins are the crucial negative regulators of immune responses that exert their effects through inhibiting the Jak/Stat signaling pathway [3]. Eight members of this family have been identified in human among them are SOCS1 and SOCS3 which alter immune responses in microglia/monocytes and astrocytes as well [4]. These two members of SOCS family are also involved in the TNF-α mediated function and signaling pathways [5, 6]. The role of *SOCS* genes in the pathogenesis of inflammatory diseases has been emphasized by the observed down-regulation of *SOCS1* and *SOCS5* genes in peripheral blood of multiple sclerosis (MS) patients [7]. However, we have recently assessed expression of *SOCS* genes in the peripheral blood of autistic patients and found no remarkable difference in their expression levels between patients and controls [8]. Based on the role of SOCS proteins in the regulation of immune responses and the observed dysregulation of immune system in BD, we hypothesized that expression of these genes are dysregulated in patients with BD. Consequently, in the present project, we examined expression levels of these genes in peripheral blood of BD patients to unravel if their transcript levels are different between patients and controls and whether they can be used as diagnostic biomarkers to differentiate BD from healthy status.

## Methods

### Study participants

Five milliliters of peripheral blood samples were collected from 50 bipolar patients and 50 healthy subjects. Patients were assessed based on the Diagnostic and Statistical Manual of Mental Disorders-5 (DSM-5) [9]. Control subjects had no past history of psychiatric or neurodegenerative diseases, mental retardation, cancer or infection. They were non-smokers and were not on any medication. All patients were under treatment with Carbamazepine and were in euthymia phase. None of patients were smokers. The study protocol was approved by ethical committee of Hamadan University of Medical Sciences. Written consent forms were obtained from all study participants.

### Expression analysis

RNA was extracted from blood samples using Hybrid-R Blood RNA (GeneAll Biotech, Korea). Afterwards, cDNA was made from RNA samples using PrimeScript 1st strand cDNA Synthesis Kit (Clontech, Japan). Relative expression of *SOCS* genes were quantified in Rotor Gene 6000 real-time PCR system using the primer and probes listed in Table 1 and the RealQ Plus Master Mix (Ampliqon, Denmark). The *HPRT1* gene was used as reference gene. As this gene is located on X chromosome, the data of males and females were analyzed separately.

**Table 1:** Nucleotide sequences of primers and probes used for expression analysis

Gene name	Primer and probe sequence	Primer and probe length	Product length
*HPRT1*	F: AGCCTAAGATGAGAGTTC	18	88
	R: CACAGAACTAGAACATTGATA	21	
	FAM -CATCTGGAGTCCTATTGACATCGC- TAMRA	24	
*SOCS1*	F: TGGCCCCTTCTGTAGGATGG	20	109
	R: GGAGGAGGAAGAGGAGGAAGG	21	
	FAM- TGGCCCCTTCTGTAGGATGG- TAMRA	20	
*SOCS2*	F: ACGCGAACCCTTCTCTGACC	20	99
	R: CATTCCCGGAGGGCTCAAGG	20	
	FAM -CTCGGGCGGCCACCTGTCTTTGC-TAMRA	23	
*SOCS3*	F: GTGGAGAGGCTGAGGGACTC	20	111
	R: GGCTGACATTCCCAGTGCTC	20	
	FAM- CACCAAGCCAGCCCACAGCCAGG- TAMRA	23	
*SOCS5*	F: GTGACTCGGAAGAGGATACAACC	23	91
	R: CTAACATGGGTATGGCTGTCTCC	23	
	FAM- CGCTGCTTCTGCCTCCGTGACTGC- TAMRA	24	

### Statistical methods

Statistical analyses were performed in the SPSS 22.0 (IBM, Chicago, IL, USA) and MedCalc statistical software. Expression levels of *SOCS* genes were compared between BD patients and normal controls using independent T test. The correlation between gene expression and demographic data of patients were assessed using regression model. Partial correlation was used to assess the strength and direction of a linear relationship between genes expressions and clinical variables (age, age at disease onset and disease duration) while controlling for the effect of gender. *P* <  0.05 was regarded as significant. The diagnostic power of transcript levels of *SOCS* genes was described by measuring the area under curve (AUC) in the receiver operating characteristic (ROC) curves. Sensitivity and specificity values were also calculated. The study power (1- β) was calculated by using Stata tool (StataCorp 2017. Stata statistical software Release 15, College station, Texas: StataCorp LLC).

## Results

### General information about study participants

Table 2 shows general demographic and clinical information of study participants.

**Table 2:** General information of study participants

Study groups	Parameters		Values
Case	Gender	Male	35
		Female	15
	Age (mean ± SD (range))		36.5 ± 9.32 (17–56)
	Age at onset (mean ± SD (range))		32.64 ± 8.04 (15–48)
	Disease duration (mean ± SD (range))		3.86 ± 2.66 (1–14)
Control	Gender	Male	35
		Female	15
	Age (mean ± SD (range))		33.62 ± 8.59 (14–52)

### Expression analysis

**Fig. 1:**
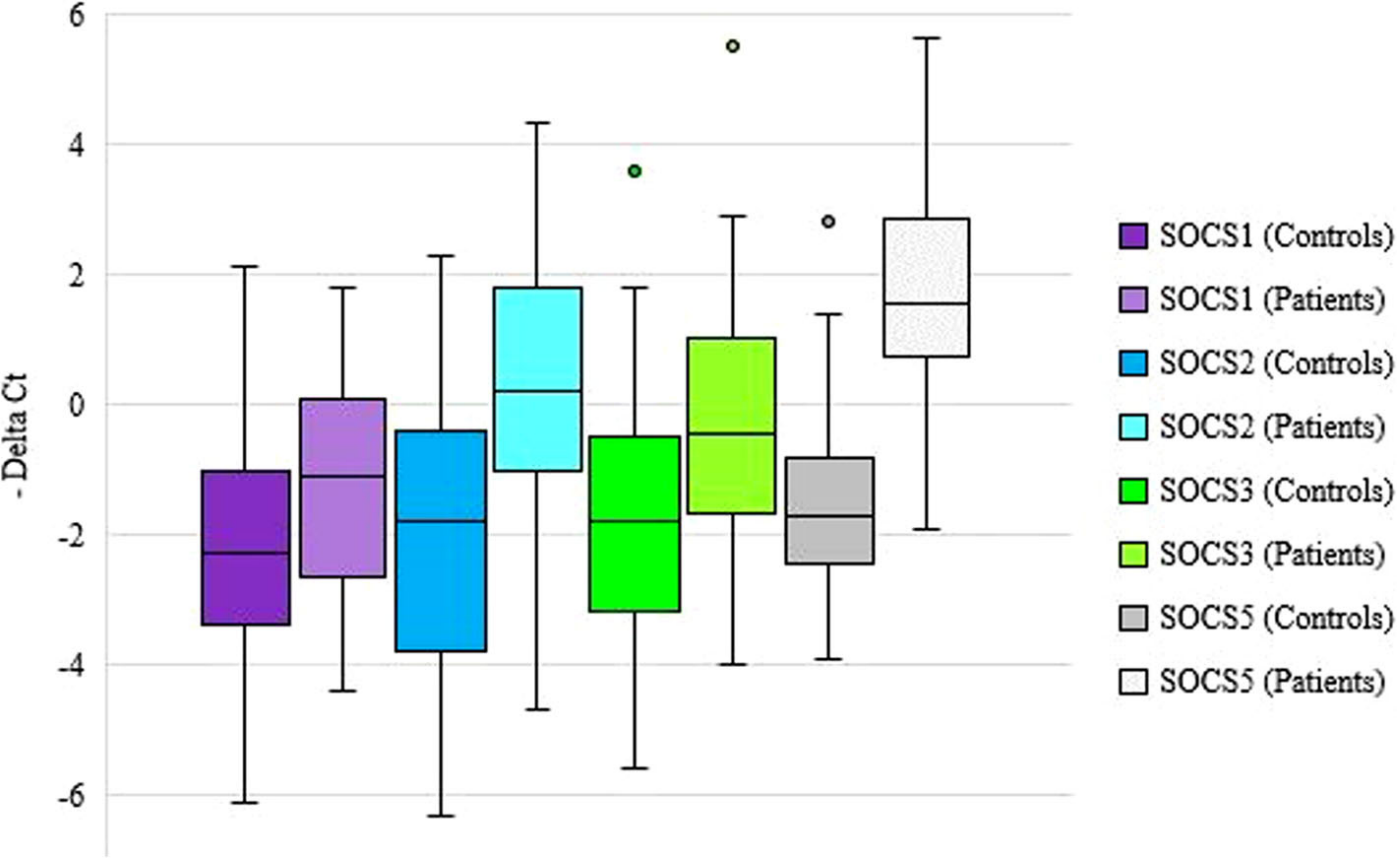
Relative expression of SOCS genes in patients and controls as demonstrated by –delta CT values (CT _reference gene_- CT _target gene_)

The power of the study (1- β) was calculated to be 82% by using Stata tool considering the mean values of *SOCS1* gene expression in cases and controls (μ1 = 1.07 (±2.02), μ2 = 2.18 (±1.86), α = 0.05). All *SOCS* genes were up-regulated in BD patients compared with healthy subjects (Fig. 1). As the selected reference gene (*HPRT1*) is located on X chromosome, the data of males and females were analyzed separately. When comparing patients with sex-matched controls, the significant differences were observed only in male subjects except for *SOCS5* which was up-regulated in both male and female patients compared with the corresponding control subjects. Table 3 shows the results of expression analysis.

**Table 3:** Relative expression of *SOCS* genes in BD patients compared with controls

Genes	Parameters	Total patients vs. total controls (50 vs. 50)	Male patients vs. male controls (35 vs. 35)	Female patients vs. female controls (15 vs. 15)
*SOCS1*	Expression ratio	2.07	4.43	0.61
	*P*-value	0.005	< 0.001	0.14
*SOCS2*	Expression ratio	3.85	8	1.21
	*P*-value	< 0.001	< 0.001	0.47
*SOCS3*	Expression ratio	2.7	4.82	1.19
	*P*-value	< 0.001	< 0.001	0.61
*SOCS5*	Expression ratio	5.77	8.77	3.81
	*P*-value	< 0.001	< 0.001	0.001

Expression levels of genes were not correlated with any of demographic or clinical parameters of study participants after adjustment for gender. Table 4 shows the results of partial correlation analysis between expression levels of genes and clinical data.

**Table 4:** Partial correlation analysis between expression levels of genes and clinical data (controlled for gender)

		*SOCS1*		*SOCS2*		*SOCS3*		*SOCS5*	
		R	*P* value	R	*P* value	R	*P* value	R	*P* value
Case	Age	0.14	0.15	0.06	0.33	0.09	0.26	0.02	0.44
	Age at onset	0.18	0.09	0.09	0.25	0.13	0.18	0.05	0.34
	Disease duration	- 0.05	0.35	- 0.07	0.31	- 0.08	0.29	- 0.1	0.24
Control	Age	- 0.04	0.38	0.19	0.08	0.1	0.23	0.06	0.33

Significant pairwise correlations were found between expression levels of genes in both patients and controls (Table 5).

**Table 5:** Pairwise correlation between expression levels of genes (R^2^ values are shown. *: Correlation is significant at *P* <  0.05 level, **: Correlation is significant at *P* <  0.01 level)

		*SOCS5*	*SOCS3*	*SOCS2*
*SOCS1*	Controls	0.43**	0.46**	0.6**
	Patients	0.28**	0. 17*	0.34**
*SOCS2*	Controls	0.51**	0.59**	
	Patients	0.67**	0.47**	
*SOCS3*	Controls	0.47**		
	Patients	0.58**		

### ROC curve analysis

**Fig. 2:**
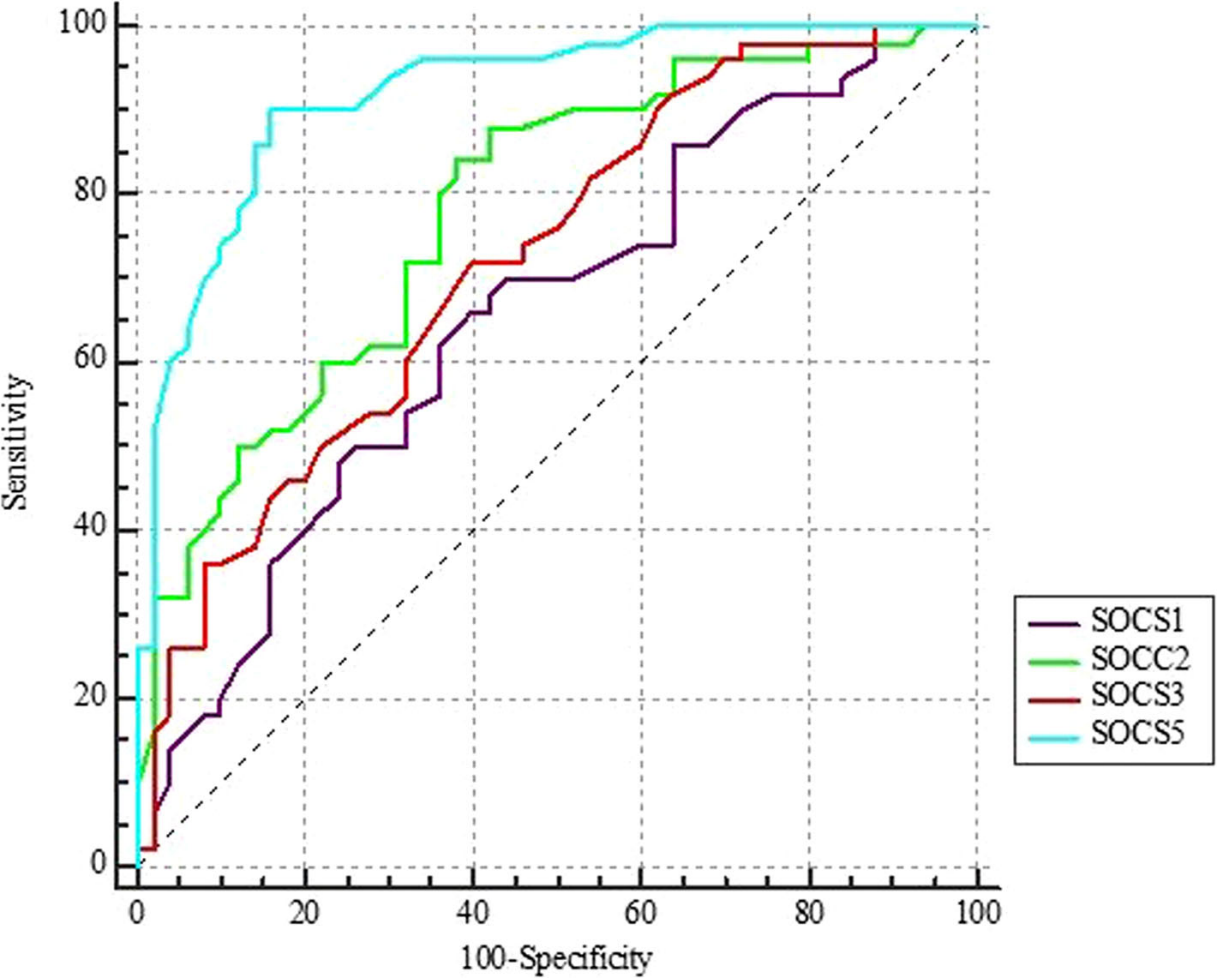
The results of ROC curve analysis for assessment of diagnostic power of *SOCS* genes in BD

Based on AUC values, SOCS5 had the best performance in differentiation of disease status in study participants (AUC = 0.92). Combination of four genes increased the specificity of tests and resulted in diagnostic power of 0.93. Table 6 and Fig. 2 show the results of ROC curve analysis.

**Table 6:** The results of ROC curve analysis (a: Youden index, b: Significance Level P (Area = 0.5), Estimate criterion: optimal cut-off point for gene expression)

	Estimate criterion	AUC	J^a^	Sensitivity	Specificity	*P*-value^b^
*SOCS1*	≤ 1.5	0.65	0.26	62	64	0.005
*SOCS2*	≤ 1.3	0.78	0.46	84	62	< 0.0001
*SOCS3*	≤ 1.3	0.72	0.32	72	60	< 0.0001
*SOCS5*	≤ 0.3	0.92	0.74	90	84	< 0.0001
Combination of four genes	> 0.54	0.93	0.8	88	92	< 0.0001

## Discussion

In the current study, we evaluated expression of *SOCS* genes in the peripheral blood of BD patients. Based on the previously reported interactions between SOCS proteins and sex steroids [10], we performed sex difference analysis as well. We found a sexual dimorphism in up-regulation of *SOCS* genes except for *SOCS5* which was up-regulated in both male and female patients compared with the corresponding control subjects. Although SOCS proteins have been primarily recognized for their anti-inflammatory functions, they might have different functions based on the tissue in which they are expressed. For instance, SOCS3 expression in neurons repressed insulin growth factor-1 (IGF-1)-induced neurite outgrowth [11] and reversed the neuroprotective effect of IGF-1 against TNF-α induced apoptosis [12]. Consistent with these functions, elevated levels of SOCS3 in oligodendrocytes and neurons after traumatic brain damage might exert harmful effects [13]. A previous study has shown decreased neurite mass in neuronal cell cultures being treated with serum of BD patients [14]. Although the underlying mechanism of such in vitro observation is not known, this study indicates the presence of specific peripheral factors that might affect central tissues of BD patients. Patel et al. have previously suggested that temporary or insistent disturbance of blood brain barrier (BBB) integrity would lead to diminished CNS defense and higher permeability of proinflammatory elements from the peripheral blood into the brain. These happenings might be involved in the pathogenesis of BD [15]. Higher levels of *SOCS* genes expression in peripheral blood of BD patients might affect integrity of BBB or might lead to higher levels of these genes in CNS tissue due to malfunctioned BBB in BD patients. Alternatively, the observed over-expression of *SOCS* genes in peripheral blood of male BD patients compared with healthy males might reflect higher levels of these genes in central tissues of BD patients as a result of a global event that modulate expression of these genes in whole tissues. On the other hand, such peripheral over-expression of *SOCS* genes might be a compensatory mechanism to alleviate the detrimental effects of cytokine over-production in BD patients. The latter is supported by the observed role of SOCS1 up-regulation in suppression of TNF-α-mediated cell oxidative stress and apoptosis [6].

Expression of SOCS proteins might also been related with levels of inflammatory markers. For instance, SOCS3 has an inhibitory effect on expression of IL-6 family cytokines [16], but promotes expression of IL-10 [17], an anti-inflammatory cytokine which is increased in serum samples of BD patients [18]. Thus, a future perspective of our work is assessment of the relationship between serum inflammatory markers and SOCS expression in BD patients and also their variations between manic and depressive patients.

SOCS3 is also involved in the evolution of leptin resistance [19]. Both leptin deficiency and leptin resistance might participate in changes in affective status [20]. Consequently, the observed higher levels of *SOCS3* in peripheral blood of BD patients might lead to leptin resistance and contribute in the pathogenesis of BD through this axis. In addition, SOCS1 and SOCS3 proteins has been shown to induce insulin [21], a condition that is associated with poor psychiatric outcomes in patients with BD [22].

Expression levels of genes were not correlated with any of demographic or clinical parameters of study participants after adjustment for gender. In our previous study in autistic patients, we found correlation between *SOCS5* expression and age of patients, but such correlation was not detected in healthy subjects [8]. However, contrary to the present study, *SOCS3* expression levels were significantly correlated with the age of all both patients and controls [8]. Taken together, the correlation between expression of *SOCS* genes and age not only depends on the disease status but is also determined by the age range. The latter is deduced from the difference in age range of study participants in our previous study versus the current study (children versus adults).

We also detected significant pairwise correlations between expression levels of *SOCS* genes in both patients and controls which further supports our previous speculation regarding the presence of a single regulatory mechanism for these genes [8].

## Conclusion

We reported dysregulation of *SOCS* genes in BD. We also assessed diagnostic power of *SOCS* transcripts in BD and found superiority of *SOCS5* as over other assessed genes. Notably, expression levels of this gene could differentiate euthymic patients from healthy subject with a diagnostic power of 0.92. Consequently, our data suggest suitability of this gene independence of patients’ signs or symptoms which potentiates it as a subsection of a panel of diagnostic biomarkers in BD in complicated situations such as in forensic medicine. However, future studies are needed to verify our results in larger sample sizes.

## Data Availability

The analysed data sets generated during the study are available from the corresponding author on reasonable request.

## Abbreviations

AUC: Area under curve
BD: Bipolar disorder
CNS: Central nervous system
DSM-5: Diagnostic and Statistical Manual of Mental Disorders-5
IGF-1: Insulin growth factor-1
MS: Multiple sclerosis
ROC: Receiver operating characteristic
SOCS: Suppressors of cytokine signaling
sTNF-R1: Soluble tumor necrosis factor receptor type 1
TNF-α: Tumor necrosis factor-α
